# Investigating cellular network heterogeneity and modularity in cancer: a network entropy and unbalanced motif approach

**DOI:** 10.1186/s12918-016-0309-9

**Published:** 2016-08-26

**Authors:** Feixiong Cheng, Chuang Liu, Bairong Shen, Zhongming Zhao

**Affiliations:** 1 Department of Biomedical Informatics, Vanderbilt University School of Medicine, Nashville, TN USA; 2 Alibaba Research Center for Complexity Sciences, Hangzhou Normal University, Hangzhou, Zhejiang China; 3 Center for Systems Biology, Soochow University, Suzhou, China; 4 Department of Cancer Biology, Vanderbilt University School of Medicine, Nashville, TN USA; 5 Department of Psychiatry, Vanderbilt University School of Medicine, Nashville, TN USA; 6 Center for Precision Health, School of Biomedical Informatics, The University of Texas Health Science Center at Houston, Houston, TX USA

**Keywords:** Cancer, Heterogeneity, Network modularity, Network entropy, Unbalanced motifs

## Abstract

**Background:**

Cancer is increasingly recognized as a cellular system phenomenon that is attributed to the accumulation of genetic or epigenetic alterations leading to the perturbation of the molecular network architecture. Elucidation of network properties that can characterize tumor initiation and progression, or pinpoint the molecular targets related to the drug sensitivity or resistance, is therefore of critical importance for providing systems-level insights into tumorigenesis and clinical outcome in the molecularly targeted cancer therapy.

**Results:**

In this study, we developed a network-based framework to quantitatively examine cellular network heterogeneity and modularity in cancer. Specifically, we constructed gene co-expressed protein interaction networks derived from large-scale RNA-Seq data across 8 cancer types generated in The Cancer Genome Atlas (TCGA) project. We performed gene network entropy and balanced versus unbalanced motif analysis to investigate cellular network heterogeneity and modularity in tumor versus normal tissues, different stages of progression, and drug resistant versus sensitive cancer cell lines. We found that tumorigenesis could be characterized by a significant increase of gene network entropy in all of the 8 cancer types. The ratio of the balanced motifs in normal tissues is higher than that of tumors, while the ratio of unbalanced motifs in tumors is higher than that of normal tissues in all of the 8 cancer types. Furthermore, we showed that network entropy could be used to characterize tumor progression and anticancer drug responses. For example, we found that kinase inhibitor resistant cancer cell lines had higher entropy compared to that of sensitive cell lines using the integrative analysis of microarray gene expression and drug pharmacological data collected from the Genomics of Drug Sensitivity in Cancer database. In addition, we provided potential network-level evidence that smoking might increase cancer cellular network heterogeneity and further contribute to tyrosine kinase inhibitor (e.g., gefitinib) resistance.

**Conclusion:**

In summary, we demonstrated that network properties such as network entropy and unbalanced motifs associated with tumor initiation, progression, and anticancer drug responses, suggesting new potential network-based prognostic and predictive measure in cancer.

**Electronic supplementary material:**

The online version of this article (doi:10.1186/s12918-016-0309-9) contains supplementary material, which is available to authorized users.

## Background

Cancer is a major public health problem in the world and approximately 25 % of deaths in the United States is due to cancer [1]. Analyses of massive amounts of cancer genomics data generated from The Cancer Genome Atlas (TCGA) and the International Cancer Genome Consortium (ICGC) has suggested that cancer is a systems-level, network phenomenon attributed to the accumulation of genetic or epigenetic alterations under molecular network architecture [2–4]. However, our understanding of cancer biology at the systems-level has still been nascent, such as genome stability or instability [5, 6]. There is an urgent need to develop network-based methods or approaches to explore systems-level, network features associated with tumor initiation, progression, and resistance of specific targeted agents so that such findings will provide new potential prognostic and therapeutic biomarkers in cancer.

Several network terms, such as “cancer network attractors” [7], “network plasticity” [8], and “network entropy” [9–12], had been proposed in cancer systems biology study. West et al. found that cancer cells often have higher network entropy by integrating microarray gene expression data into a protein interaction network (PIN) [9]. Banerji et al. suggested that signaling entropy provided a potential measure in cancer by investigating microarray gene expression data in 3668 breast cancer samples and 1692 lung adenocarcinoma samples [10]. Therefore, network entropy can be a useful quantitative measure to characterize different disease status, like tumor versus normal tissue as well as various stages of progression.

In molecularly targeted cancer therapeutics, the most common approach is to find molecules that can directly lead to the death of cancer cells, such as kinase inhibitors. However, targeted agents (e.g., kinase inhibitors) often develop high risk of drug resistance due to the feedback or crosstalk signaling mechanisms within cellular networks [13]. One possible reason is that currently targeted therapy often introduces stress and further lead to increase the degree of heterogeneity of a cancer cell population in the long therapeutic period despite short-term induction of cancer cell death [14]. The end result will be to speedup the process of drug resistance through cancer evolution. Furthermore, normal cells will be at a survival disadvantage as they are much less dynamic than cancer cells. However, our understanding of the systems-level network features that characterize anticancer drug responses has been largely behind the clinical practice in cancer fields.

**Fig. 1:**
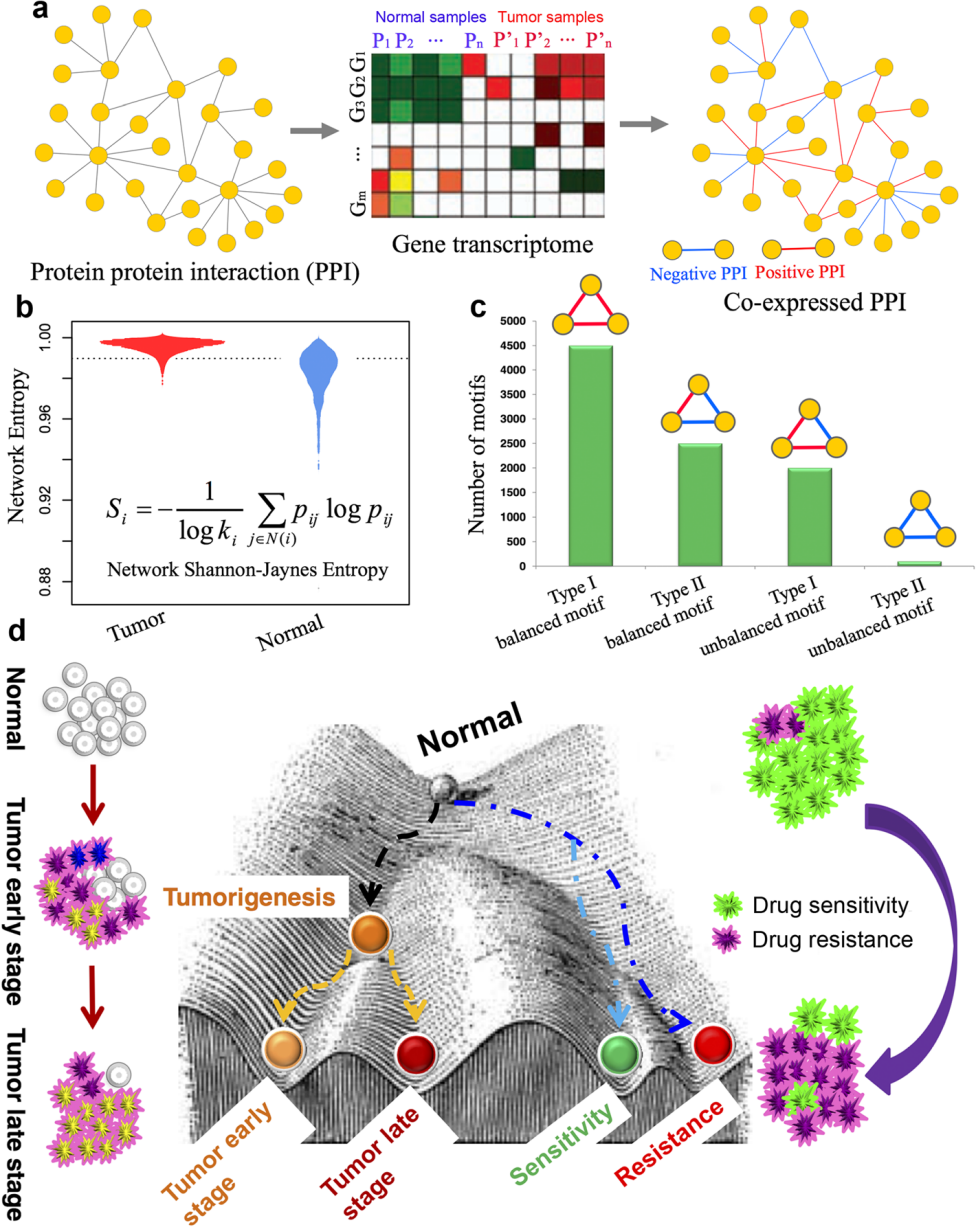
Diagram of the network entropy and unbalanced motif approach to quantify cancer cellular network heterogeneity and modularity. **a** Workflow for gene co-expressed protein interaction network construction using large-scale RNA-Seq data from The Cancer Genome Atlas. Network entropy (**b**), and **c** balanced versus unbalanced motif analysis. **d** Quantifying cancer cellular network heterogeneity and modularity characterized by network entropy, and balanced versus unbalanced motifs in tumor versus matched normal samples, different stages of tumor progression, and anticancer drug sensitive versus resistant cancer cell lines. Based on the notion of Waddington’s landscape [17], network entropy and ratio of unbalanced versus balanced motifs were represented as the energy potential in Waddington’s landscape. High cellular network heterogeneity during tumorigenesis is characterized by elevated network entropy and high ratio of unbalanced versus balanced motifs

In this study, we proposed an integrated network-based framework to examine whether network properties (e.g. network entropy or unbalanced motifs) are associated with tumor initiation and progression, and anticancer drug responses (Fig. 1). Specifically, we built each co-expressed PIN (CePIN) to describe specific cellular network statuses characterizing tumorigenesis, progression (four stages), and anticancer drug responses by integrating the large-scale RNA-Seq data across 8 cancer types from TCGA and the microarray gene expression data from the Genomics of Drug Sensitivity in Cancer (GDSC) database [15, 16]. We then employed network entropy and balanced versus unbalanced motif approaches to quantitatively characterize tumor initiation, progression, and anticancer drug responses. Using network entropy as measure, we found that cells could be characterized by an increase in both network entropy and unbalanced motifs during tumorigenesis. Moreover, we could use the network entropy to quantify various stages of progression and anticancer drug responses. In summary, this study would provide new potential network-based prognostic and predictive biomarkers in cancer.

## Results

### Overview of a network entropy and unbalanced motif approach

In this study, we proposed a network-based entropy and unbalanced motif approach based on the notion of Waddington’s landscape [10, 17] as shown in Fig. 1. First, we built each gene CePIN by incorporating RNA-Seq V2 data covering tumor and normal tissue samples across 8 cancer types from TCGA into a high-quality PIN (Fig. 1a). These 8 cancer types consist of breast invasive carcinoma (BRCA), colon adenocarcinoma (COAD), head and neck squamous cell carcinoma (HNSC), kidney renal clear cell carcinoma (KIRC), lung adenocarcinoma (LUAD), lung squamous cell carcinoma (LUSC), thyroid carcinoma (THCA), and uterine corpus endometrial carcinoma (UCEC). We next built each CePIN to characterize anticancer drug sensitive versus resistant cancer cell lines using microarray gene expression and drug pharmacological data from GDSC database [15, 16]. We then performed network analysis, such as network entropy and balanced versus unbalanced motifs in each CePIN (Fig. 1b and c). Finally, we systematically examined cellular network heterogeneity and modularity through aforementioned network measures in tumors versus normal tissue samples, various stages of tumor progression, and anticancer drug resistant versus sensitive cancer cell lines, respectively (Fig. 1d). Based on the notion of Waddington’s landscape (Fig. 1d), we used network entropy and a ratio of unbalanced versus balanced motifs as the energy potential in Waddington’s landscape [10, 17]. Thus, we speculated that network entropy and the ratio of unbalanced versus balanced motifs can be used to quantitatively characterize cellular network heterogeneity and modularity in cancer. For instance, highly cellular network heterogeneity during tumorigenesis is marked by high network entropy and high ratio of unbalanced versus balanced motifs.

### Increased cellular network entropy during tumorigenesis

**Fig. 2:**
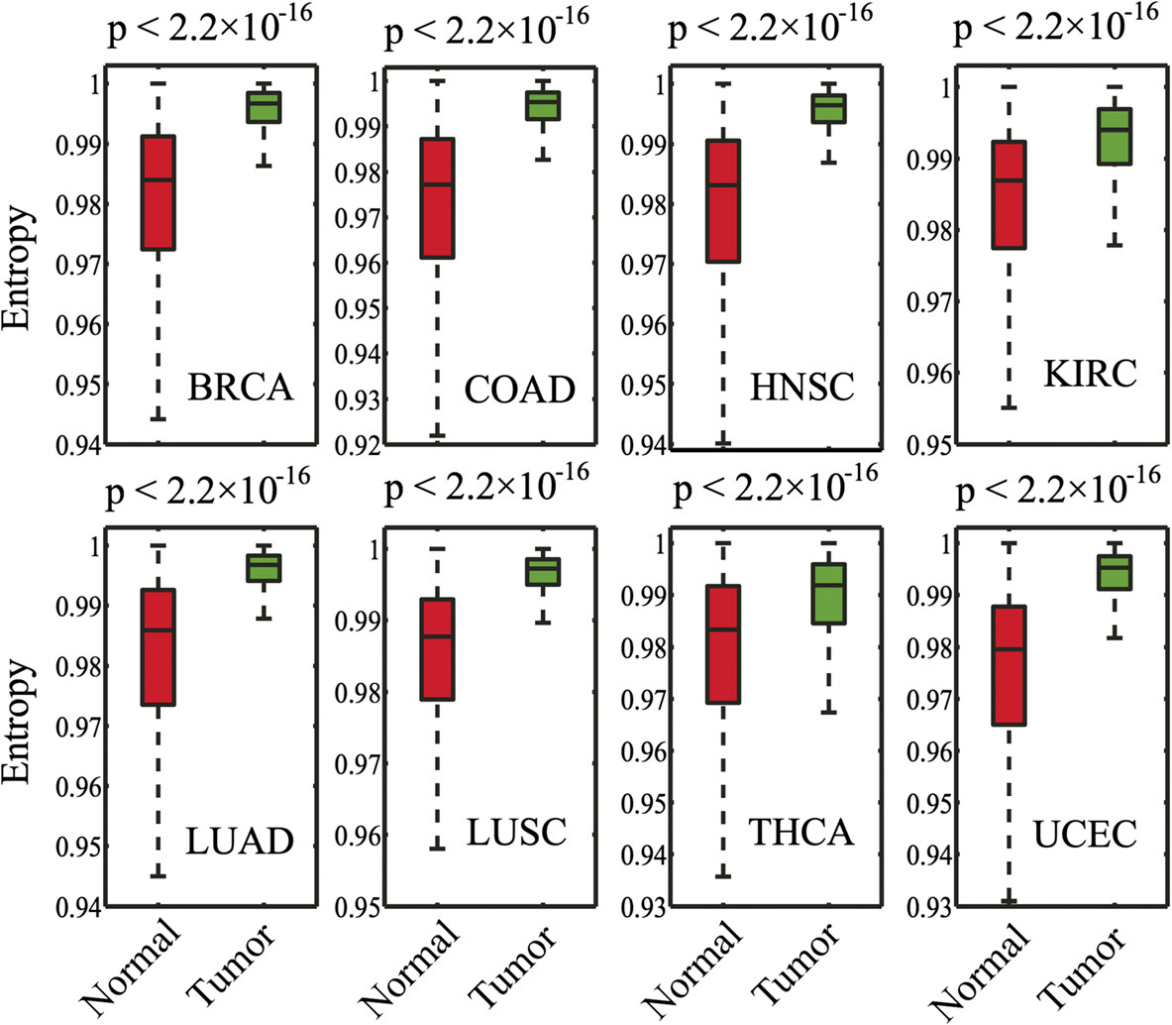
Boxplots showing the distribution of genome-wide (~10,000 genes) local network entropy between tumor samples and normal tissue samples for 8 cancer types. Breast invasive carcinoma (BRCA), colon adenocarcinoma (COAD), head and neck squamous cell carcinoma (HNSC), kidney renal clear cell carcinoma (KIRC), lung adenocarcinoma (LUAD), lung squamous cell carcinoma (LUSC), thyroid carcinoma (THCA), and uterine corpus endometrial carcinoma (UCEC). The p-value was calculated by Wilcoxon rank-sum test

We collected and processed the RNA-Seq V2 data (Additional file 1: Table S1) for tumor and normal tissue samples in 8 cancer types from TCGA, since only these 8 cancer types had at least 10 tumor samples and 10 normal samples. A good number of samples in gene expression data are required to build the reliable CePIN and to perform the follow up analyses. We calculated Pearson Correlation Coefficient (PCC) using RNA-Seq V2 data for each cancer type and available normal tissue samples, and then mapped PCC to large-scale PIN to build CePIN (Fig. 1a). Each CePIN contains ~100,000 edges and ~10,000 genes. We then calculated network entropy for each gene node in CePIN based on a previous study [9] (see Materials and Methods). We first examined genome-wide (~10,000 genes) local network entropy between tumor and normal tissues. As shown in Fig. 2, we found that tumors had higher genome-wide local network entropy compared to that of normal tissues in all of the 8 cancer types (*p* < 2.2 × 10^−16^, Wilcoxon rank-sum test). There was minor variation of network entropy for each gene as shown in Fig. 2, consistent with several previous studies [9–11].

**Fig. 3:**
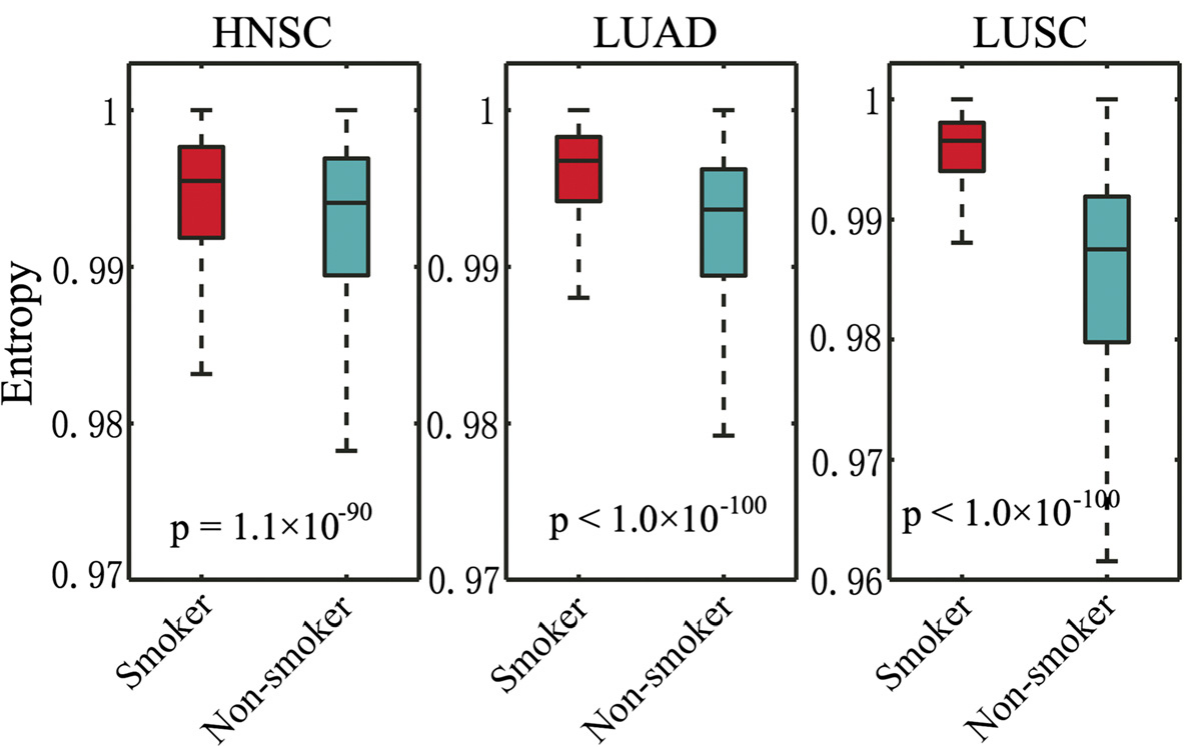
Boxplots showing the distribution of genome-wide local network entropy between smokers and non-smokers in 3 cancer types. Lung adenocarcinoma (LUAD), lung squamous cell carcinoma (LUSC), and head and neck squamous cell carcinoma (HNSC). The p-value was calculated by Wilcoxon rank-sum test

We next examined genome-wide local network entropy distribution for 8 normal tissue types and 8 cancer types, respectively. Among the 8 normal tissues, colon had the lowest average genome-wide local network entropy (0.9670 ± 0.0003), while lung squamous had the highest average genome-wide local network entropy (0.9822 ± 0.0002), as shown in Fig. 2. The observation of different local network entropy in different normal tissues might be due to tissue differentiation during development [10, 18]. However, Fig. 2 showed that different cancer types had heterogeneous genome-wide local network entropy distribution compared to that of 8 normal tissues. Among the 8 cancer types, four: LUSC (0.9957 ± 0.0001), LUAD (0.9950 ± 0.0001), BRCA (0.9947 ± 0.0001), and HNSC (0.9945 ± 0.0001), showed the highest average local network entropy distribution. The observation of high local network entropy in breast cancer might be explained by its high tumor heterogeneity [19]. For LUAD, LUSC, and HNSC, some environmental factors, such as smoking, may promote tumor heterogeneity and accordingly, cause higher network entropy. A previous study revealed that an average somatic mutation frequency in smokers was more than 10-fold higher in never-smokers in non-small cell lung cancer [20]. To test this hypothesis, we further separated TCGA patients into smokers and never-smokers in LUAD, LUSC, and HNSC, and rechecked the genome-wide local network entropy distribution. Figure 3 shows that smokers are characterized by a higher network entropy compared to that of non-smokers in all of the 3 smoking-related cancer types: LUAD (*p* < 1.0 × 10^−100^), LUSC (*p* < 1.0 × 10^−100^), and HNSC (*p* = 1.1 × 10^−90^).

### An increase of cellular network unbalanced motifs during tumorigenesis

We next studied the network structural balance theory using our data. Specifically, we examined the ratio of unbalanced versus balanced motifs to quantify the stability of a network structure in a given condition (e.g., tumors versus normal tissues) [21, 22]. As shown in Table 1, similar to social networks, normal tissues had more balanced motifs than that of tumors. Importantly, tumors had more unbalanced motifs than that of normal tissues in all of the 8 cancer types we examined. Unbalanced motifs are particularly interesting because they are highly dynamic and unstable [23]. For example, type II unbalanced motifs (Fig. 1c), consisting of two positive and one negative gene-gene interactions, should potentially be recognized as negative feedback loops or incoherent feed-forward loops. These two kinds of loops are both associated with adaptation responses and may be crucial for tumor cellular network system controllability.

**Table 1:** Distribution of balanced versus unbalanced motifs in tumor samples and normal tissues in 8 cancer types

Cancer type	Fraction of balanced motifs		Fraction of unbalanced motifs	
	Normal	Tumor	Normal	Tumor
BRCA	0.913	0.835	0.087	0.165
COAD	1.00	0.919	0.000	0.081
HNSC	0.994	0.909	0.006	0.091
KIRC	0.948	0.819	0.052	0.181
LUAD	0.979	0.879	0.021	0.121
LUSC	0.979	0.877	0.021	0.123
THCA	0.984	0.845	0.016	0.155
UCEC	1.00	0.980	0.000	0.020

### Characterizing tumor progression by network entropy

**Fig. 4:**
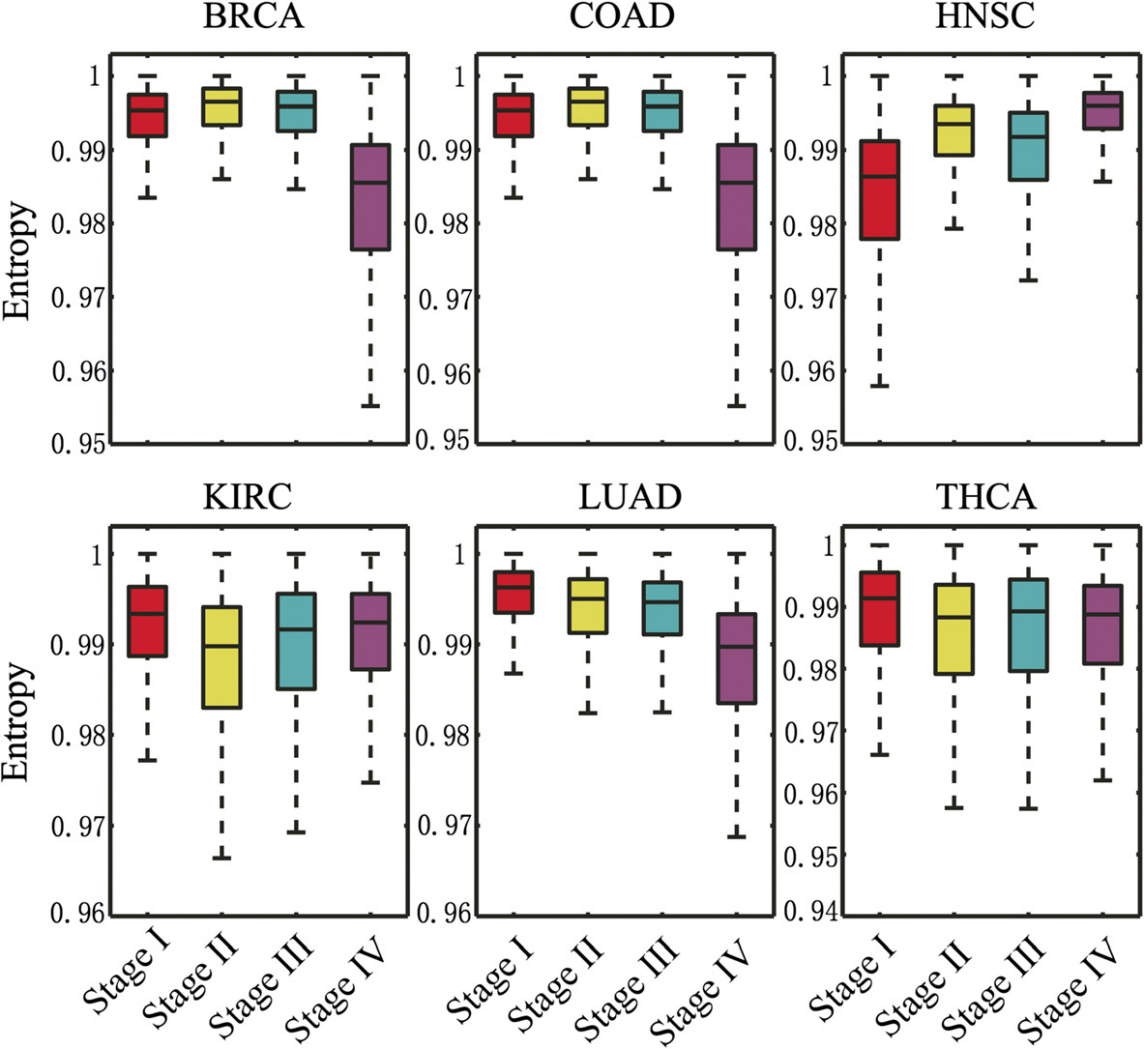
Boxplots showing the distribution of genome-wide local network entropy across 4 stages (I-IV) of tumor progression in 6 cancer types. Abbreviations of 6 cancer types are provided in Fig. 1’s legend

We next investigated whether network entropy is associated with different stages of tumor progression. We collected the available RNA-Seq V2 data across 4 stages (I to IV) in 6 cancer types from TCGA, since only 6 cancer types had sufficient number (>10 samples) of samples in each stage for building gene CePIN (Additional file 1: Table S2). Figure 4 revealed that different tumor stages showed heterogeneous distribution of the cellular network entropy. For BRCA, COAD, and LUAD, stage IV had a lower genome-wide local network entropy compared to that of stages I (*p* < 0.01, Wilcoxon rank-sum test), II (*p* < 0.01) and III (*p* < 0.01). In contrast, stage IV in HNSC had a higher genome-wide local network entropy compared to that in its stages I (*p* < 2.2 × 10^−16^), II (*p* < 0.01) and III (*p* < 0.01). THCA and KIRC only showed a minor genome-wide local network entropy changes across 4 different stages. Low network entropy observed in stage IV (metastasis) compared to that of low stages (stages I to III) might be explained by tumor clonal evolution [24]. For example, during tumor subclonal evolution (e.g., a clonal sweep), a new clone that took over the entire population and replaced the ancestral clones would result in a homogeneous cell population with low network entropy during tumor metastasis (e.g., breast cancer) [24, 25].

We next compiled a list of high-quality, significantly mutated genes (SMGs) in cancer from 4 pan-cancer genomic analysis projects, as described in our previous study [6]. A total of 614 SMGs were collected. Similar to the genome-wide network entropy analysis, BRCA and LUAD indicated the lowest network entropy for SMGs in stage IV (*p* < 0.01) compared to that in stages I-III (Additional file 1: Figure S1). HNSC had the highest network entropy for SMGs in stage IV compared to that in stages I-III (*p* < 0.01). We next examined Cancer Gene Census (CGC) genes, which are well curated and have been widely used as a reference cancer gene set in various cancer-related studies [26, 27]. Similar trends were observed for CGC genes compared to that in SMGs and genome-wide analysis (Additional file 1: Figure S2). We further collected 477 oncogenes (OGs) and 1040 tumor suppressor genes (TSGs) from our previous study [6, 28], and then examined the network entropy value for OGs and TSGs. We found a similar network entropy distribution for OGs and TSGs compared to that for genome-wide genes, SMGs, and CGC genes across 4 stages in the 6 cancer types (Additional file 1: Figures S3 and S4). Taken together, our results suggested that different stages of tumor progression might be characterized by heterogeneous network entropy distribution for both genome-wide and cancer-related genes: SMGs, CGC genes, OGs, and TSGs.

### Characterizing anticancer drug responses by network entropy

We next investigated whether a subset of cancer cell lines showing different anticancer drug responses (e.g. sensitivity or resistance) could be characterized by dynamic network entropy. We compiled normalized microarray gene expression data and drug pharmacological data on cancer cell lines from the GDSC database [15, 16]. We separated the cancer cell lines into two subsets (resistant and sensitive) based on drug maximal screening concentration described in two previous studies [15, 16]. In order to reliably estimate covariance of two genes across a set of cancer cell lines, we selected cancer types that had at least 10 cell lines with drug responses and microarray gene expression data simultaneously. Based on this criterion, we compiled four molecularly targeted drugs (Gefitinib, Dasatinib, Nilotinib, and Temsirolimus) in 4 cancer types of cell lines (lung, breast, blood, and skin) from the GDSC database.

**Fig. 5:**
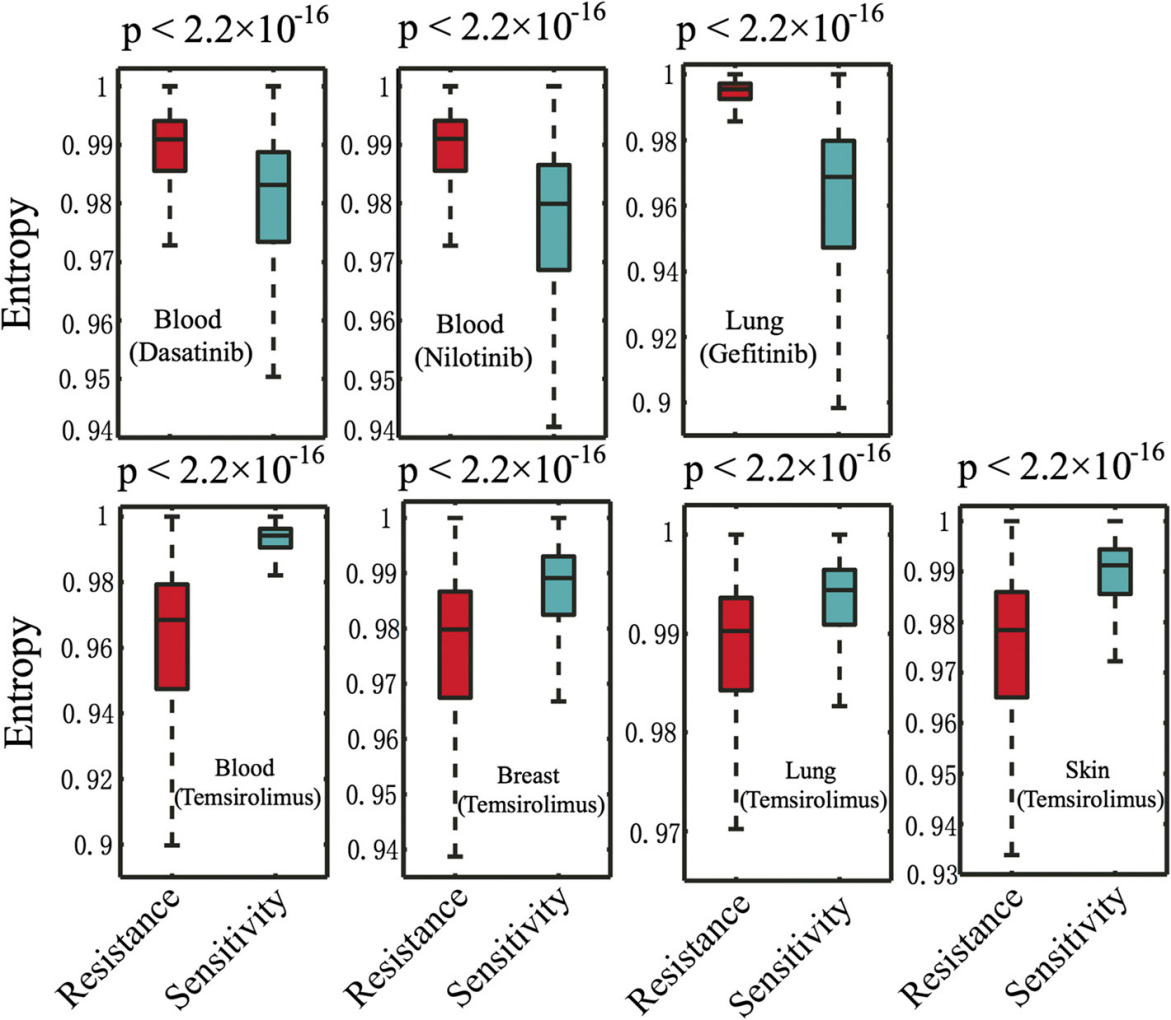
Boxplots showing the distribution of genome-wide local network entropy between drug resistant and sensitive cancer cell lines for 4 targeted anticancer drugs in 4 types of cancer: blood, lung, breast, and skin. Local network entropy distribution between drug resistant and sensitive cancer cell lines for 458 drug-sensitivity genes was provided in Additional file 1: Figure S5. The p-value was calculated by Wilcoxon rank-sum test

Figure 5 showed that 3 tyrosine kinase inhibitors (Gefitinib, Dasatinib, and Nilotinib) resistant cell lines had a higher genome-wide local network entropy compared to that of their sensitive cell lines in blood and lung cancer (*p* < 2.2 × 10^−16^, Wilcoxon rank-sum test). However, serine/threonine protein kinase inhibitor (Temsirolimus) resistant cell lines had a lower genome-wide local network entropy compared to that of the sensitive cell lines in all of the 4 cancer types: lung, breast, blood, and skin cancer, as shown in Fig. 5. We further compiled 458 genes that were involved in sensitivity or resistance of 130 anticancer drugs from a previous study [16]. As shown in Additional file 1: Figure S5, similar network entropy distribution was observed for 458 drug-sensitivity genes compared to that in genome-wide local network entropy analysis (Fig. 5).

## Discussion

### Smoking is associated with cancer cellular network heterogeneity and drug responses

Previous studies revealed that some environmental factors like smoking were likely to increase heterogeneity within a tumor [29, 30]. We examined the network entropy distribution in a set of tumor samples that included both smoking and non-smoking histories. Figure 3 revealed that smokers had higher genome-wide network entropy compared to that of non-smokers in all of the 3 smoking-related cancer types: LUAD, LUSC, and HNSC. In our recent studies, we also found that smokers had unique mutation signatures [31] and higher mutational heterogeneity using a mathematical model [6]. Collectively, environmental factors, such as smoking, may play crucial roles during tumorigenesis and often speedup cancer cellular network heterogeneity. Furthermore, the increased cancer cellular network heterogeneity caused by smoking may be associated with drug responses. For example, Fig. 5 showed that Gefitinib resistant lung cancer cell lines were often characterized by elevated network entropy. Filosto et al. suggested that cigarette smoke mediates lung cancer development and resistance to tyrosine kinase inhibitors (e.g. Gefitinib) [32]. Kim et al. found that lung cancer patients with more than 30 pack-years smoking dosage have significantly poorer survival outcome on tyrosine kinase inhibitor therapy [33]. Collectively, this study suggested potential network-level evidence that smoking may increase cancer cellular network heterogeneity and further contribute to tyrosine kinase inhibitor resistance. Hence, our network analysis yielded a potential network-based predictor for quantitatively characterizing the clinical outcome of molecularly targeted treatment (e.g. tyrosine kinase inhibitor) in lung cancer.

### Limitations and future directions

There are several potential limitations in current study. For example, we used large-scale RNA-Seq data across four stages of tumor progression in 6 cancer types from TCGA to study tumor progression quantified by network entropy. However, we did not observe a consistent pattern of network entropy distribution in four different stages of progression across 6 cancer types. One possible reason is that most of the current TCGA projects only generated RNA-Seq data for primary tumors. Although different tumor stage information was annotated for TCGA samples, most tumor samples sequenced in TCGA were collected from primary tumors, not metastatic tumors, limiting the accuracy of our network entropy analysis as shown in Fig. 4. Thus, further investigations are warranted for systematically examining the network entropy based on data sequenced in both primary and metastatic tumors, which we hope will be prompted by the findings herein. For example, a recent TCGA study sequenced 266 metastases and 67 primary cutaneous melanomas [34]. This will provide more useful datasets to examine melanoma cellular network heterogeneity in the future.

In addition, although we used a large-scale PIN for network entropy and unbalanced motif analysis, current network analyses also have some limitations, such as network incompleteness and possible data noise. For example, current PPI networks identified by high-throughput technologies may only cover less than 20 % of all potential pairwise PPIs in the human cells [35, 36]. PPIs are tissue or cell type specificity. However, we assembled all PPIs from different tissues or cell types as a global background in this study, which may cause potential data bias. In addition, the unbalanced number in tumors, normal tissues, and different tumor stages (Additional file 1: Table S1) may also influence the results. For example, the number of stage IV BRCA samples was 15, much less than the numbers of BRCA samples in stages I, II and III (Additional file 1: Table S2). While the number of stage IV LUAD sample (22) was smaller that that normal lung tissue samples (58), the network entropy in stage IV LUAD was higher than that of normal lung tissues (*p* < 0.01). This result suggested that the unbalanced number of tumor samples during tumor progression might not influence the overall conclusion in this study. Finally, ultra-mutated tumor samples in some cancer types such as colon cancer may also influence the analysis result. For instance, a small set of tumor samples can contribute to a large proportion (e.g., up to 40 %) of total somatic mutations observed in the whole cancer cohort [37]. Figure 4 revealed that stage IV had the lowest genome-wide network entropy distribution compared to that of stages I-III in COAD. However, we did not observe a similar trend of network entropy distribution for four cancer-related gene sets: SMGs, CGC genes, OGs, and TSGs, in COAD.

## Conclusion

In this study, we proposed a network entropy and unbalanced motif approach to systematically investigate network features during tumor initiation, progression, and anticancer drug responses quantified by cellular network heterogeneity and modularity under the notion of Waddington’s landscape. We found that tumorigenesis was characterized by increased network entropy and unbalanced motifs compared to that of normal tissues using TCGA data. Furthermore, the increased network entropy may be associated with anticancer drug resistance. In a case study, we found that smoking is characterized by the increased cellular network heterogeneity, suggesting potential network level evidence associated with tyrosine kinase inhibitor (e.g. Gefitinib) resistance induced by smoking in lung cancer. In addition, different stages of tumor progression are characterized by highly heterogeneous network entropy, which may contribute to high risk of drug resistance in the molecularly targeted cancer therapy. In summary, this study could provide new potential network-based predictively prognostic and therapeutic biomarkers for cancer systems biology study and the molecularly targeted cancer therapeutics.

## Methods

### Construction of protein interaction network

We constructed a high-quality PIN covering 113,473 unique interactions connecting 13,579 protein-coding genes based on our previous studies [6, 13, 28, 38]. We implemented three data cleaning steps to select a high-quality PPI pair. First, we only compiled a high-quality PPI pair if it was experimentally validated in human models through a well-defined experimental protocol. Second, we re-annotated all protein-coding genes using gene Entrez ID and the gene official symbols from the National Center for Biotechnology Information (NCBI) database [39]. Finally, self-loop interactions or duplicated PPI pairs were excluded. The detailed data collection and preparation are provided in our previous studies [6, 13, 28].

### Preparation of RNA-Seq data and gene co-expression analysis

We downloaded RNA-Seq V2 data from 3557 tumor samples across 8 cancer types and 418 matched normal tissues from TCGA (October 02, 2013) [40]. These 8 cancer types consisted of BRCA, COAD, HNSC, KIRC, LUAD, LUSC, THCA, and UCEC (Additional file 1: Table S1). In this study, we implemented two steps to define the genes that were expressed: (i) in a sample, we filtered out a gene whose mRNA expression was below the 20 % of all mRNAs ordered by their expression level; and (ii) we further filtered out a gene that expressed in less than 20 % of samples in the whole expression matrix. We also extracted RNA-Seq V2 data for smokers and never-smokers in LUAD, LUSC, and HNSC from TCGA (January 05, 2015) using the R package implemented in TCGA-Assembler [41]. In addition, we collected normalized microarray gene expression data for drug sensitive versus resistant cancer cell lines from the GDSC database (July 01, 2014) [15, 16]. Finally, we calculated PCC value for each gene-gene pair and mapped PCC value of each gene-gene pair onto aforementioned PIN to construct CePINs for cancer types (including different stages of tumor progression), normal tissues, smokers versus non-smokers, and drug sensitive versus resistant cancer cell lines, respectively (Fig. 1a).

### Network entropy analysis

In this study, we denoted *PCC*_*ij*_ as PCC value of a gene co-expression pair between gene *i* and *j* in PIN. Since − 1 ≤ *PCC*_*ij*_ ≤ 1, the edge weights of CePIN can be redefined as ${w}_{ij}=\frac{1}{2}\left(1+PC{C}_{ij}\right)$ based on two previous studies [9, 12].

For each gene *i*, we calculate the local Shannon-Jayne entropy as follows:

$$ {S}_i=-\frac{1}{ \log \left({k}_i\right)}{\displaystyle \sum_{j\in N(i)}}{p}_{ij} \log \left({p}_{ij}\right) $$

Where *k*_*i*_ is the number of gene *i*’s neighbors, *N*(*i*) is the set of gene *i*’s neighbors in CePIN and ${p}_{ij}=\frac{w_{ij}}{{\displaystyle {\sum}_{j\in N(i)}}{w}_{ij}}$ is the proportion of gene *i*’s total weights that links to gene j.

We quantified cancer cellular network heterogeneity as a function of the network entropy under on the notion of Waddington’s landscape [10, 17], which can be represented as the distribution of local entropies across the whole network.

### Balanced versus unbalanced motif analysis

Relations between genes on CePIN often reflect a mixture of positive and negative PCC value (negative versus positive PPI in the right panel of Fig. 1a). The interplay between positive and negative relationships significantly affects the network structure. In this study, the signed triangle motifs on three genes are extracted from CePIN, where the edge *ij* in the triangle motif is signed as positive when *PCC*_*ij*_ > 0, and negative when *PCC*_*ij*_ < 0. There are four types of signed triangle motifs (See Fig. 1c). Following the classical structural balance theory [42], the motifs with odd number (1 or 3) of positive edges are more plausible, which are considered as the balanced motifs (balanced motif Type I and Type II in Fig. 1c), while the motifs with even number (0 or 2) of positive edges are considered as the unbalanced motifs (unbalanced motifs Type I and Type II in Fig. 1c). And the balanced motifs should be more prevalent in stable systems.

Here, the proportion of the unbalanced motifs (*p*_*um*_) could be used to represent the disorder level of the network structure:

$$ {p}_{um}=\frac{N_{um}}{N_{tm}} $$

where, *N*_*um*_ is the number of the unbalanced motifs in the network, and *N*_*tm*_ is the total number of the triangle motifs in the network. The large *p*_*um*_ value shows the more heterogeneous network structure, and we can detect the evolution of the various states for different cancer types by comparing the *p*_*um*_ value. To perform reliably balanced versus unbalanced motif analysis, we only kept the significantly co-expressed pairs having p-value < 0.05 (F-statistics) in each CePIN for unbalanced versus balanced motif analysis.

### Cancer gene sets

We collected four cancer-related gene sets: 614 cancer SMGs, 487 CGC genes, 477 oncogenes, and 1040 TSGs, as briefly described in our previous study [28]. The abbreviations of these gene sets were described in the Results section. We further compiled 458 genes that were involved in sensitivity or resistance of 130 anticancer drugs from two previous studies [13, 16]. In that study, the authors comprehensively identified drug-sensitivity genes on 639 human tumor cell lines using the integrated genomics analysis [16].

### Statistical analysis

All statistical tests were performed using the R package (v3.0.1) [43].

## Additional file

Additional file 1:
**Table S1.** The statistics of RNA-Seq data for tumors and normal tissues in 8 cancer types collected from TCGA. **Table S2.** The statistics of RNA-Seq data for four stages of tumor progression in 6 cancer types collected from TCGA. **Figure S1.** Local network entropy distribution for cancer significantly mutated genes among four stages (I-IV) of tumor progression in 6 cancer types. **Figure S2.** Local network entropy distribution for Cancer Gene Census (CGC) genes among four stages (I-IV) of tumor progression in 6 cancer types. **Figure S3.** Local network entropy distribution for oncogenes (OGs) among four stages (I-IV) of tumor progression in 6 cancer types. **Figure S4.** Local network entropy distribution for tumor suppressor genes (TSGs) among four stages (I-IV) of tumor progression in 6 cancer types. **Figure S5.** Local network entropy distribution for 458 drug-sensitivity genes in drug sensitive versus resistant cancer cell lines. (DOCX 994 kb)

## Abbreviations

CePIN: Co-expressed Protein Interaction Network
ICGC: International Cancer Genome Consortium
OG: Oncogene
PCC: Pearson Correlation Coefficient
PIN: Protein Interaction Network
PPI: Protein-Protein Interaction
TCGA: The Cancer Genome Atlas
TSG: Tumor Suppressor Gene
