# Supplementary material for: Investigating cellular network heterogeneity and modularity in cancer: a network entropy and unbalanced motif approach
Source: BMC Syst Biol. 2016 Aug 26;10(Suppl 3):65. doi: 10.1186/s12918-016-0309-9 (PMC5009528; doi:10.1186/s12918-016-0309-9)
Supplement: Additional file 1: — Table S1. The statistics of RNA-Seq data for tumors and normal tissues in 8 cancer types collected from TCGA. Table S2. The statistics of RNA-Seq data for four stages of tumor progression in 6 cancer types collected from TCGA. Figure S1. Local network entropy distribution for cancer significantly mutated genes among four stages (I-IV) of tumor progression in 6 cancer types. Figure S2. Local network entropy distribution for Cancer Gene Census (CGC) genes among four stages (I-IV) of tumor progression in 6 cancer types. Figure S3. Local network entropy distribution for oncogenes (OGs) among four stages (I-IV) of tumor progression in 6 cancer types. Figure S4. Local network entropy distribution for tumor suppressor genes (TSGs) among four stages (I-IV) of tumor progression in 6 cancer types. Figure S5. Local network entropy distribution for 458 drug-sensitivity genes in drug sensitive versus resistant cancer cell lines. (DOCX 994 kb) [file 12918_2016_309_MOESM1_ESM.docx]

S5: Feixiong Cheng, Chuang Liu, Bairong Shen and Zhongming Zhao. Investigating cellular network heterogeneity and modularity in cancer: a network entropy and unbalanced motif approach

| **Authors** | | | | | | |
| --- | --- | --- | --- | --- | --- | --- |
| first name | last name | email | country | organization | Web site | corresponding? |
| Feixiong | Cheng | feixiong.cheng@vanderbilt.edu | United States of America | Vanderbilt University |  |  |
| Chuang | Liu | liuchuang1985@126.com | China | Hangzhou Normal University |  |  |
| Bairong | Shen | bairong.shen@suda.edu.cn | China | Soochow University |  |  |
| Zhongming | Zhao | zhongming.zhao@vanderbilt.edu | United States of America | Vanderbilt University |  | ✔ |

Reviewer:

| Jun | Kong | jun.kong@emory.edu | Emory University |
| --- | --- | --- | --- |
| Ge | Gao | gaog@mail.cbi.pku.edu.cn | Peking University |
| Kun | Huang | kun.huang@osumc.edu | OSU |
| Dmitry | Korkin | korkin@missouri.edu | University of Missouri |

Summary of Received Reviews and Comments

Reviews superseded by other reviews are shown in the grey color in the table. All times are GMT.

|  | date | PC member | subreviewer | Overall evaluation | Reviewer's confidence | 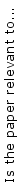 | 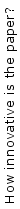 | 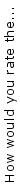 | 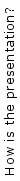 | 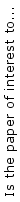 |  |
| --- | --- | --- | --- | --- | --- | --- | --- | --- | --- | --- | --- |
| [Review 1](https://www.easychair.org/conferences/review_for_paper.cgi?a=8929944;paper=2450453#Review_3808571) | Aug 25 | Jun Kong |  | **2** | **4** | 3 | 4 | 5 | 5 | 4 |  |
| [Review 2](https://www.easychair.org/conferences/review_for_paper.cgi?a=8929944;paper=2450453#Review_3824138) | Sep 2 | Ge Gao |  | **1** | **4** | 3 | 3 | 4 | 4 | 4 |  |
| [Review 3](https://www.easychair.org/conferences/review_for_paper.cgi?a=8929944;paper=2450453#Review_3845008) | Sep 12 | Kun Huang | Hao Ding | **1** | **3** | 3 | 2 | 4 | 4 | 3 |  |
| [Review 4](https://www.easychair.org/conferences/review_for_paper.cgi?a=8929944;paper=2450453#Review_3853331) | Sep 17 | Dmitry Korkin |  | **3** | **4** | 3 | 5 | 4 | 4 | 4 |  |

Reviews and Comments

| **Review 1** | |
| --- | --- |
| PC member: | Jun Kong |
| Overall evaluation: | **2**: (accept) |
| Reviewer's confidence: | **4**: (high) |
| Is the paper relevant to ICIBM?: | 3: (Yes) |
| How innovative is the paper?: | 4: (good) |
| How would you rate the technical quality of the paper?: | 5: (excellent) |
| How is the presentation?: | 5: (excellent) |
| Is the paper of interest to users and practitioners?: | 4: (Yes) |
| Review: | This paper proposes to use a network entropy of Pearson Correlation Coefficients of gene pairs and unbalanced motif (motifs with even number of positive edges) to quantify cellular network heterogeneity and modularity during tumor initiation, progression, and anticancer drug responses under the notion of Waddington’s landscape. It shows that increased network entropy and unbalanced motifs could suggest tumorigenesis. Therefore, this study provides a potential way to study cancer biology and targeted therapeutics with network-based prognostic and therapeutic biomarkers.  This paper is very well written. Experiments are well designed. Results are clearly demonstrated to support the findings. Following is a list of minor suggestions that may be useful to improve the paper further.  1 Please correct “PPC” to “PCC” in “Increased cellular network entropy during tumorigenesis” under Results section.  2 Is there any reason in literature that explains why the network entropy for certain stage IV tumor (e.g. BRCA) is lower than that of lower stage? Could this possible suggest network entropy may not be a selective indicator for high stage tumor?  3In Table S2, the number of IV BRCA samples is 15, much less than number of BRCA samples in I, II, and III stage. Given this fact, is it possible that network entropy of tumor BRCA samples would be lower than that of normal BRCA samples if we had a larger number of Stage IV BRCA samples?  4 In “Methods and Materials” section, the Shannon-Jayne entropy for a gene is defined. However, the way to compute the network entropy of a given set of genes is not clear.  Overall, this is a great work. |
| Confidential remarks for the program committee: |  |
| Time: | Aug 25, 23:50 |

| **Review 2** | |
| --- | --- |
| PC member: | Ge Gao |
| Overall evaluation: | **1**: (weak accept) |
| Reviewer's confidence: | **4**: (high) |
| Is the paper relevant to ICIBM?: | 3: (Yes) |
| How innovative is the paper?: | 3: (fair) |
| How would you rate the technical quality of the paper?: | 4: (good) |
| How is the presentation?: | 4: (good) |
| Is the paper of interest to users and practitioners?: | 4: (Yes) |
| Review: | By systematically analyzing transcriptome profiles of eight cancers, the authors inferred co-expression protein-interaction networks, and further examined two network-level properties (i.e. network entropy and ratio of balanced vs unbalanced motifs) at multiple contexts. The manuscript is generally well written but there are still a few issues which should be addressed before publishing:  First of all, while these identified patterns themselves are interesting, an additional (and fine-scale) discussion of the underling (biological) mechanisms would be very helpful for further investigation (e.g. how the change of network entropy could contribute to/be connected to tumor progression/drug response?)  Moreover, it would be great if the authors could also give p-value(s) for their several statements involved Fig 4, Fig S1-4, esp. would the apparent differences be statistically significant (or not)?  The last but not the least, the authors should give a detailed list of (accession numbers of) transcriptome samples they analyzed, maybe as a suppl. table, so that others can evaluate/check their results when necessary. |
| Confidential remarks for the program committee: |  |
| Time: | Sep 02, 13:58 |

| **Review 3** | |
| --- | --- |
| PC member: | Kun Huang |
| Reviewer: | Hao Ding <ding.96@osu.edu> |
| Overall evaluation: | **1**: (weak accept) |
| Reviewer's confidence: | **3**: (medium) |
| Is the paper relevant to ICIBM?: | 3: (Yes) |
| How innovative is the paper?: | 2: (poor) |
| How would you rate the technical quality of the paper?: | 4: (good) |
| How is the presentation?: | 4: (good) |
| Is the paper of interest to users and practitioners?: | 3: (Maybe) |
| Review: | This manuscript presented a network entropy and unbalanced motif approach to  examine cellular network heterogeneity and modularity in multiple types of  cancer. This is a relevant topic that fits the remit of the ICIBM.  The co-expression network construction, entropy and motif analysis are plausible. The authors also conducted the analysis on a fairly comprehensive data set. The manuscript is well written. However, the methods and material are not novel.  Considering that the innovation of this paper is limited, it is important that  the implication of the study really stands out. The main result of this  manuscript is that tumorigenesis was characterized by increased network entropy  and unbalanced motifs compared to that of normal tissues. The similar results  has been reported in [1]. In addition, the manuscript mention that the network  analysis could yield a potential predictor for quantitatively characterizing the clinical outcome of molecularly targeted treatment. I would like to see more  description on how this potential predictor can be developed.    [1] Zhang, Jie, et al. "Weighted frequent gene co-expression network mining to  identify genes involved in genome stability." (2012): e1002656. |
| Confidential remarks for the program committee: |  |
| Time: | Sep 12, 21:05 |

| **Review 4** | |
| --- | --- |
| PC member: | Dmitry Korkin |
| Overall evaluation: | **3**: (strong accept) |
| Reviewer's confidence: | **4**: (high) |
| Is the paper relevant to ICIBM?: | 3: (Yes) |
| How innovative is the paper?: | 5: (excellent) |
| How would you rate the technical quality of the paper?: | 4: (good) |
| How is the presentation?: | 4: (good) |
| Is the paper of interest to users and practitioners?: | 4: (Yes) |
| Review: | This is an interesting work that aims in developing a network-based approach to investigate different stages of cancer by comparing network heterogeneity and modularity.  The paper is well-structured and organized. I only have a few concerns to address:  p. 13: "First, we only compiled a high-quality PPI pair if it was experimentally validated in human models through a well-defined experimental protocol."   What are those well-defined experimental protocols? Large-scale Y2H? TAP-MS? Others that are low-throughput?  p.14: "These 8 cancer types consisted of BRCA, COAD, HNSC, KIRC, LUAD, LUSC, THCA, and UCEC"  Please provide the actual names for the cancer types (e.g., BRCA=breast cancer).  P. 14: "this study, we implemented two steps to define the genes that were expressed: (i) in a sample, we filtered out a gene whose mRNA expression was below the 20% of all mRNAs ordered by their expression level; and (ii) we further filtered out a gene that expressed in less than 20% of samples in the whole expression matrix."  What is a rationale for those preprocessing steps? Have those thresholds been used before? |
| Confidential remarks for the program committee: |  |
| Time: | Sep 17, 04:23 |
